# Traumatic arteriovenous fistula as consequence of TMJ arthroscopic surgery. A case report

**DOI:** 10.4317/jced.52954

**Published:** 2016-07-01

**Authors:** Paolo Cariati, Ana-Belen Marin-Fernandez, Fernando Monsalve-Iglesias, Maria Roman-Ramos, Blas Garcia-Medina

**Affiliations:** 1 Oral and Maxillofacial surgery resident. Hospital Universitario Virgen de las nieves, Granada, Spain; 2 Maxillofacial Surgeon. Hospital Universitario Virgen de las nieves, Granada, Spain

## Abstract

The ocurrence of a traumatic arteriovenous fistula after arthroscopic surgery of TMJ represents an extremely rare event. Specifically, this uncommon complication has been described only in a few case reports. In this light, the most frequent symptoms showed by this disease are thrills, bruits, pulsatile tinnitus, and an expansible vascular mass. Importantly, the severity of these symptoms is also dependent on the vessels involved. With regard to the management, is important to note that the vessel ligation with surgery as well as vessel emolization with endovascular procedures have been shown to be effective in the treatment of these cases. In view of that, the present study describes a case of superficial temporal arteriovenous fistula that arose as a postoperative complication of a bilateral arthroscopic eminoplasty of TMJ. The aim of the present report is to characterize this rare syndrome with the goal of proposing suitable treatments.

** Key words:**Arteriovenous fistula, arthroscopic surgery, eminoplasty of TMJ, temporal vessels.

## Introduction

Arteriovenous fistula are abnormal communications between arteries and veins (1). Consequently, these condition could cause a pathological arteriovenous shunt. These disorders may be congenital, spontaneous, or traumatic (2). In this line, trauma is the most frequent cause of arteriovenous fistulas (2). Notwithstanding, the occurrence of a traumatic fistula after arthroscopic surgery of TMJ is an uncommon event (3). In fact, only few case reports described this rare complication (3). For instance, Sacho *et al.* reported a case of arteriovenous fistula of the middle meningeal artery as complication of TMJ arthroscopic surgery (3). Furthermore, Calwell also described a case of arteriovenous fistula that appeared in the postoperative of TMJ arthroscopic surgery (3).

According to the literature the indications for treatment include pain, hemorrhage, pressure symptoms, ischemic ulceration, and high-output cardiac failure (3). More in detail, this arthroscopic complication might be treated using endovascular procedures or with the ligation of the malformation through open surgery (2). However, nowadays, there is no evidence about the advantages of one treatment over another.

## Case Report

**Figure 1:**
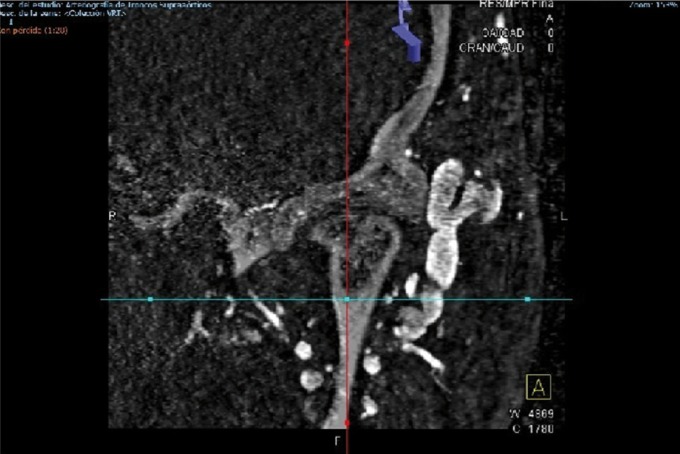
Arteriovenous fistula of superficial temporal vessels as consequence of arthroscopic eminoplasty of TMJ.

**Figure 2:**
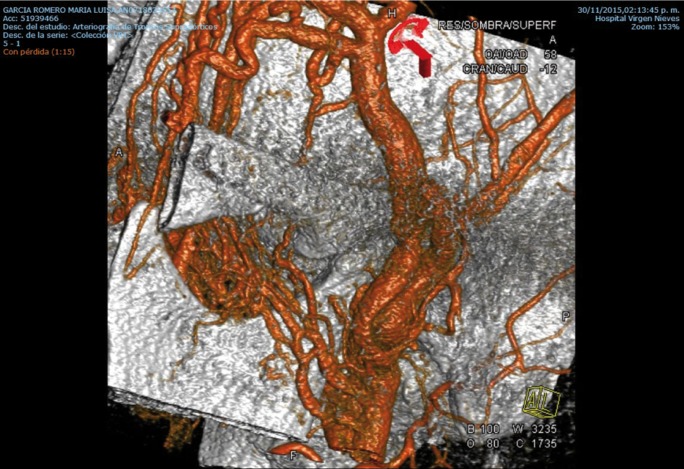
Arteriovenous fistula between superficial temporal artery and vein.

**Figure 3:**
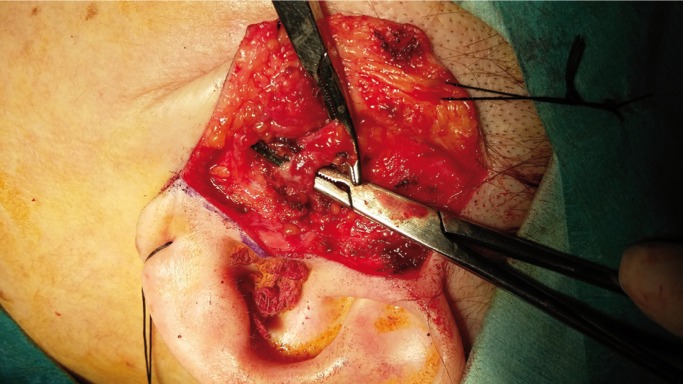
Intraoperative appearance of fistula.

We describe the case of a 46-year-old woman who underwent bilateral arthroscopic eminoplasty of TMJ. In fact, the patient suffered TMJ internal deragement. Interestingly, no problems were reported during the surgery. Despite these endeavours, we could observe the typical signs of an arteriovenous fistula 2 weeks after surgery. In this light, the patient reported local pain, bruit and sensation of expanding pulsating mass (left side). Thus, we suspected that the patient were likely to suffer a superficial temporal arteriovenous fistula as a consequence of TMJ arthroscopic eminoplasty. With this idea in mind, we decided to perform a diagnostic angiogram of the head. Finally, this test showed the existence of an iatrogenic arteriovenous fistula involved the superficial temporal artery and surrounding venous outlets. In view of the foregoing, we decide to treat the patient with open surgery through a preauricular approach. More deeply, we would like to emphasize that we took this decision due to the long waiting list of interventional neuroradiology service of our Hospital. In this sense, we would to stress that the surgery was highly successful. Indeed, the symptoms reported by patient disappeared after the surgical ligation of fistula. Lastly, the patient was discharged from hospital with a check-up appointment in 6 months to monitor the clinical evolution. It is important to highlight that during this check-up, we observed that the patient remained asymptomatic, (Figs. 1-3).

## Discussion

Currently, arthroscopic surgery is revolutionizing the classic management of some TMD. In fact, this technique is associated with a low complication rate (3). Specifically, several authors reported that arthroscopic approach shows lower postoperative morbidity and faster postoperative recovery time with respect to other TMJ surgical approach (4). However, arthroscopic surgery is not free of complications. Indeed, the numerous articular components, the facial nerve and the superficial temporal vessels may be involved in the development of iatrogenic injuries (5). Nevertheless, only few papers reported the ocurrence of an iatrogenic arteriovenous fistulas complicating TMJ arthroscopic surgery (3). In this context, we affirm that, in spite of the great number of TMJ underwent to arthroscopic surgery in our center (more than 700), we have never found this complication before. Even so, there is no evidence in the literature of arteriovenous fistula reported after a TMJ arthroscopic eminoplasty. Interestingly, the most frequently affected vessel is the superficial temporal artery because of its location on the lateral and superficial side of the TMJ capsule (6). Regarding the treatment, the review of the literature shows that this complication might be treated with the ligation of the malformation through open surgery (2). Nonetheless, some authors referred that greats result could be achieved with the embolization of arteriovenous fistula with endovascular procedures (7). In light of the above, we want to underline that we decide to treat the patient with the ligation of the proximal feeders and distal efferent outflow vessels using open surgery (preauricular approach). Specifically, this decision was taken due to the long waiting list for performing the endovascular embolization of malformation. Moreover, we would like to point out that surgery was highly effective in closing the fistula. Indeed, the recurrence of the arteriovenous fistula was not evidencied during the patient follow-up. Concluding, we focus on two major point. Firstly, special care should be taken not only during the realization of the intraarticular maneuvers, but also during the insertion of the arthroscopic cannulas. More in depth, the proximity of the temporal vessels regarding to the position of postero-lateral cannula might explain this consequence. In fact, we firmly believe that this complication occurs during the placement of this cannula. Second, altough arthroscopic TMJ surgery may show specific risk we firmly bilieve that it could be regarded as a safe surgical procedures.
